# The roles of age, gender, and migration in shaping adolescent student satisfaction within Chilean schools

**DOI:** 10.1038/s41598-024-61427-2

**Published:** 2024-06-17

**Authors:** Cristian Céspedes, Camila Leigh, Enrique Leigh, Peodair Leihy, Sergio Fuentealba-Urra, Andrés Rubio, Damarys Roy

**Affiliations:** 1 Andres Bello University, Faculty of Economics and Business, Santiago, Chile; 2 Centro de Investigación Urbana para el Desarrollo, el Hábitat y la descentralización (CIUDHAD), Universidad Andrés Bello, Santiago, Chile; 3 Universidad Del Desarrollo, Santiago, Chile; 4 Diego Portales University, Faculty of Psychology, Santiago, Chile

**Keywords:** Migrant education, Student satisfaction, Age factors, Gender factors, Acculturation, Human behaviour, Climate-change mitigation

## Abstract

This study has a quantitative cross-sectional design that aims to investigate the relationships between gender, age, status (migrant or Chilean-born), educational satisfaction, and overall life satisfaction among adolescent students in 7th and 8th grades of the Chilean educational system. The sample includes 406 students from four municipal public educational centers located in the Santiago district of the Metropolitan Region of Chile, with at least 20% migrant enrollment. The data were analyzed using quantitative methods using the R language, with descriptive analysis, cross-tabulation analysis, and independence tests. The packages used were: ggplot2, tidyverse and ggstatplot. The study found that age has an impact on the level of satisfaction with education and relationships formed with peers. Specifically, younger students expressed higher levels of satisfaction in these areas compared to older students. Moreover, foreign students showed a statistically significant difference in relation to age range, suggesting that as these students grow older, they tend to become less satisfied with the relationships they form with their classmates. In contrast, no statistically significant difference was found among Chilean students, indicating that the findings discussed here may not be generalizable to this specific population. The analysis also indicates a significant difference in the entire sample, suggesting a correlation between age range and level of satisfaction with schooling.

## Introduction

From the turn into the twenty-first century, Chile has experienced an unprecedented inflow of immigrants, overwhelmingly from other parts of Latin America. The Chilean economy would boom from the late 1980s until towards the end of the 2010s, eventually becoming a magnet for migrants with differing educational levels from often troubled countries to the north^1^. Chile’s own broader social tensions would boil over in October 2019^2^. This would set in train a redoubled commitment to create a new constitution reflecting contemporary aspirations including more functional socioeconomic equity, gender relations and cultural pluralism^3^. A winsome constitutional proposal with populist intent was forthrightly rejected in September 2022. One stumbling block remains a disorienting tension between internationalist pretenses and middle-class perceptions that the state does not sufficiently favor Chileans over migrants through its meager welfare provisions^4^.

The recently established trend of net inward migration appears to have taken root, although developments for the remainder of the 2020s look set to depend on the relative stability of different parts of the region^4,5^. Meanwhile, statistics from the early 2020s also point to tens of thousands of young adult Chileans leaving in search of better opportunities. This is an unusual scenario, in the context of a much-depressed birth rate and an aging demographic profile, Chile appears to need to shore up its future workforce and also to foster a more creative and trusting culture, which in immigrant-infused contexts typically calls for an education system capable of promoting greater social cohesion. It is a learning process for a palpably diversifying country^6^.

Throughout the nineteenth and much of the twentieth century, the Republic of Chile attracted some degree of European and Middle Eastern migration, slowing to a crawl during the long military dictatorship 1970s and 80s; from the 1990s, Perú became the main source of immigrants, with other waves emerging from Bolivia, Colombia and Ecuador^7,8^, and more recently Venezuela and Haiti^9^. With increased immigration, Chile faces a gamut of related issues, including integration into wider society, and issues of equity and human rights^10–12^ Table 1 lays out the accelerating growth in immigrant numbers:

**Table 1:** Number of immigrants in Chile up to 2022.

Period of entry	Number	As proportion of previous number
Before 1990	48,521	
1990–1999	63,450	130%
2000–2009	136,603	215%
2010–2017	500,132	266%
2018–2021	1482,390	296%

Source: INE, 2022.

As of December 31, 2021, the countries of Venezuela (30%), Peru (16.6%), Haiti (12.2%), Colombia (11.7%), and Bolivia (8.9%) accounted for the majority of foreign nationals registered as residents in Chile. The proportion of males (744,213) to females (738,177) was somewhat higher. Between the ages of 25 and 39 made up the majority of adult immigrants for both sexes, with the 30–34 age group having the highest proportion of immigrants overall (18%). There were 909,414 immigrants in the Santiago-centered Metropolitan Region, making up around 12% of the total population. The mining-focused Antofagasta Region (106,274) came in second, followed by the Valparaiso Region (97,058), which is close to Santiago.

The Metropolitan Region has a high net migration rate (that is, proportion of incomers minus those having left the jurisdiction, in both cases including Chilean transmigrants), even though the most recent census from 2017 has quickly become obsolete given immigration trends. At the local level, Santiago Central had the most net migration, at 30.2%, followed by Recoleta and Estación Central, two nearby communes, at 15.6% and 27.8%, respectively (INE, 2018). Due to this circumstance, the percentage of students enrolled in the Chilean school system who are either immigrants or the offspring of immigrants is significantly higher than it was in 2015—from 0.9% to 3.2% of all students—with a rise in foreign-born enrollment of more than 300% between 2015 and 2018^13^,in Santiago Centro the rise would be from 8.9% in 2015 to 15.5% in 2018.

Chilean policy and public discourse often closely harmonizes with the international normative framework rooted in the Universal Declaration of Human Rights; important corollaries guiding the approach to migration and schooling include the International Covenant on Economic, Social and Cultural Rights^14^ and the International Convention on the Rights of the Child^15^.With regard to the latter, states sovereignly commit to deliver and respect rights related to the best interests of the child, his/her survival, development and protection, non-discrimination, and their participation in decisions affecting them. Two-time Chilean president Michelle Bachelet (2006–2010; 2014–2018) worked particularly closely with the UN, welcoming recommendations made by its Committee on the Rights of the Child^16^ regarding improvements in access to education and inclusion as well as measures to regularize the recognition of children of foreigners, allowing them clearer access to the health system.

Obviously there can persist a discrepancy between recognition of rights, and this studies on a longer standing watching brief as to the subjective living conditions of children and adolescents, notably as developed in the Spanish-speaking world by Spaniard Ferrán Casas^17^. This integral approach has been an important part of the observance of the Convention on the Rights of the Child in Chile.

^18^ argues that quality of life can be observed and measured by considering different material and emotional conditions, whether positive or negative. Material macro-objective variables often labeled (not least in self-consciously developing countries) as development, progress and economic growth, are hereby complemented by individual variables such as subjective well-being and happiness, as experienced and reported by individuals themselves^19–21^.

Regarding migrant students’ school satisfaction, various studies have staked out the phenomenon through multifactorial constructs with positive correlations. One such study in the Chilean context reveals that there are significant relationships between migrant status and self-esteem towards mathematical ability, as well as overall achievement in favor of migrant students even when this group perceives higher levels of discrimination^22^. Again in Chile, Haitian-origin school students (many of whose parents often face linguistic difficulties in Spanish) face differential perceptions from Chilean peers: Haitian-origin girls in the first four years of primary school are positively regarded by peers, whereas a significant negative bias tends to develop towards to Haitian-original males through the fifth to eighth years of school cycle^23^. Elsewhere in Latin America, discrimination and racism are revealed as components of how migrant-background children and adolescents describe daily life and in other statistical records of their interactions with others^24^.

Overall, constructs of classroom climate, life satisfaction, academic self-concept and quality of life in Chilean schools indicate a positive and mutually reinforcing relationship between these more specific substrands^25,26^. Further recent research indicate that schools reproduce and even amplify positive and negative aspects of wider society are reproduced, making them key and necessary sites for public policies are a key setting for interventions that promote public policies for integration and inclusion, and overcoming the intersectional compounding of iniquity^27^.

Regarding the construct of subjective well-being, there are two fundamental timeframes: emotional, affective aspects related to mood patterns, and cognitive and evaluative aspects to chronic life satisfaction^28,29^. Therefore, considering global life satisfaction as part of subjective well-being, the idea of incorporating a monoitem scale of global life satisfaction when studying subjective well-being was already supported^30^, since global life satisfaction is a cognitive component in which the individual reports the evaluation of his/her life as a whole with respect to perspectives and expectations that the same individual has set for him/herself^31^.

In this sense, subjective well-being has three distinctive features: (1) its subjective nature, since it refers to the individual's own assessment of her or his life; (2) its all-encompassing perspective, since a cognitive evaluation of life as a whole takes place; and (3) the necessary inclusion of positive measures, rather than simply vetting for the absence of negative aspects^32,33^.

A distinction was developed^34^ (obviously drawing on philosophical traditions going back to Socrates and Plato) within subjective well-being between two basic approaches, namely, the hedonic conception, which relates subjective well-being to the sensation life satisfaction, and the eudaimonic conception, which relates subjective well-being to achievements and goal attainment. In turn, other schools have usefully relayed this dichotomy into Spanish-language analysis; we would add that eudaimonia in this sense can be thought of as a more objective or socially negotiated kind of subjectivity^35^.

In this context, it is traditionally considered that there are certain constructs that contribute positively to subjective well-being. Among these constructs, relationships with family members and others stand out, as well as social support^36–39^. On the other hand, there are components such as age that are negatively related to subjective well-being, as demonstrated by a series of studies^20,31,40–42^.

Now, the need for indicators other than a global assessment of life satisfaction was crucial for Cummins himself and collaborators to form the International Wellbeing Group (IWbG)^43^ and create the PWI (Personal Well-being Index) and establish other domains of subjective well-being, namely: standard of living, health, achievements, relationships with the environment and security both personal and in the future.

Adolescent migration is a complicated and multifaceted phenomenon that can have a significant impact on many aspects of adolescents' lives, including overall life satisfaction and school satisfaction. The recent rise in adolescent migrants settling in Chile has sparked significant interest, particularly concerning their integration and overall quality of life. This growing demographic shift touches on crucial aspects such as education, health, and general well-being, highlighting the need for comprehensive support mechanisms^8,9,23,44^.

With the country witnessing an uptick in young migrants, it's crucial to tailor policies and interventions to meet their unique needs, considering the nuanced roles of age and gender in this dynamic^45,46^.

School satisfaction is essentially how students perceive their school life, encompassing the learning environment, relationships with educators and peers, and the quality of support and instruction received. It's about understanding the impact of these perceptions on students' academic success, social integration, and emotional health^47–50^. Studies suggest that students who are more satisfied with their school experience tend to achieve better academically, adapt more easily socially and emotionally, and enjoy greater mental health^51^. For migrant youth, navigating a new educational system and fitting into unfamiliar academic surroundings can present significant challenges, potentially influencing their satisfaction levels.

Life satisfaction, on the other hand, represents an individual's overall contentment with life, encompassing various domains such as work, family, health, finances, and personal relationships. It's about how people assess their lives in terms of their material, social, and emotional well-being^52,53^. Higher life satisfaction is often linked to better physical and mental health outcomes, academic achievement, and the quality of social interactions. Interestingly, studies have shown that migrant youths in Chile may experience lower life satisfaction than their non-migrant peers, primarily due to the hurdles they face in adapting to their new surroundings^44^. However, the experience of migration and its impact on life satisfaction can greatly vary across different contexts.

Contrastingly, other investigations have found no marked differences in life satisfaction between immigrant and native-born adolescents within the same setting^54,55^. It's important to note that the interplay between gender, migration, life satisfaction, and school satisfaction is likely to be influenced by the specific context.

The gender disparity in life satisfaction is a complex issue, shaped by various factors including cultural norms, societal expectations, experiences of discrimination, and access to resources^56–58^. Similarly, gender differences in school satisfaction between Chilean and migrant adolescents have been observed. For example, research indicates that adolescent girls, both Chilean and migrant, might report lower levels of school satisfaction compared to boys, a phenomenon influenced by a range of factors such as gender-based discrimination, academic engagement, and the social climate within schools^51,59,60^.

Thus migrant youth may struggle to get an education, adjust to a new school system, and integrate into a new academic environment. These difficulties may have an impact on student satisfaction.

Life satisfaction is a multifaceted construct that reflects an individual's subjective assessment of their life and how they value their material, social, and emotional well-being. Job satisfaction, family life, health, finances, and personal relationships are among the factors considered^52,53^. According to research, higher life satisfaction is associated with improved physical and mental health, academic performance, and the quality of social relationships. Several studies have found that migration has an impact on youth life satisfaction. It has been discovered that Chilean youth migrants have lower life satisfaction than non-migrant youth due to difficulties adjusting to their new environment^44^. It is important to state that the migration experience may greatly vary depending on contexts.

Other studies in the same context, however, have found no significant differences in life satisfaction between immigrant and native-born adolescents^54,55^. Gender, migration, life satisfaction, and school satisfaction findings may be context sensitive.

However, the reasons for these gender differences in life satisfaction may be complex and multifaceted, influenced by factors such as cultural norms, social expectations, discrimination, and access to resources^56–58^. Gender differences in school satisfaction between Chilean and migrant adolescents have also been reported in some studies. For instance, some research has found that female adolescents, both Chilean and migrant, may report lower levels of school satisfaction compared to males. This could be influenced by various factors, including gender-based discrimination, differences in academic performance or engagement, and social dynamics within the school environment^51,59,60^.

In Chile, the findings obtained by the International Survey on Children's Well-being (ISCWeB), using the PWI scale, highlight health and material goods as the most relevant factors when explaining subjective well-being in a sample of 2734 boys and girls, aged 8, 10 and 12 years, residents of the three main urban areas of Chile, even above interpersonal relationships^61^. Similar studies in adolescents^62^ observe that the highest scores in terms of subjective well-being are related to satisfaction with family, safety and home, followed by material satisfaction. In this same line, it was determined that material goods, health and family relationships could explain the subjective well-being of adolescents^19^.

Regarding studies comparing subjective well-being and overall life satisfaction between the native and migrant populations, countries such as Spain have more experience in them and several studies have determined that the native population in general has better indices of subjective well-being and overall life satisfaction compared to the migrant population with respect to a series of structural and psychosocial variables^63^. In relation to studies in this line in the adolescent population^64^, found no significant differences between Spanish native adolescents and migrant adolescents in terms of global satisfaction with life, only finding statistically significant differences in terms of sex, with males scoring higher than females. In the Chilean school context, there are no studies available that contrast subjective well-being and global life satisfaction between native adolescents and migrant adolescents, even though different social and cultural contexts have a particularly important impact on childhood and adolescence^31,33^.

Despite some studies and advances, subjective wellbeing is an incipient area that still needs to be deepened in different areas^61^ and research that measures subjective wellbeing in the adolescent population taking into account the migration variable, given that there is no such research in the Chilean context.

In light of the unstable political-social conditions in Latin America that have led to large-scale human displacements to Chile, concerns have been raised about discrimination and vulnerability faced by migrants^10^. As a country that receives migrants and is committed to human rights conventions, Chile has a responsibility to promote the welfare of its population within a democratic and tolerant environment.

Against this backdrop, questions have arisen about the levels of well-being and life satisfaction among Chilean and migrant students, with a particular focus on gender and age differences. The aim of this research is to examine the level of subjective well-being and global life satisfaction among a sample of students in the Commune of Santiago, Chile, with attention paid to their Chilean or migrant status, as well as their gender and age. Additionally, the study aims to identify the domains of subjective well-being that explain global life satisfaction in this sample. Given the growing number of immigrant adolescents in Chile and the unique challenges they face, it is crucial to understand the factors that contribute to their school satisfaction and life satisfaction, and to identify areas of intervention that can improve their academic performance and well-being.

## Methodology

This study has a cross-sectional quantitative design^65^ used in cohorts. For these purposes the cohort concept was employed: "if the population to be sampled has a common experience or characteristic which defines the sampling (e.g. all born in the same year), it is known as a cohort study”^66^. The research aims to investigate the relationships between gender, age, status (migrant or Chilean-born), educational satisfaction, and overall life satisfaction among adolescent students in 7th and 8th grades of the Chilean educational system. The sample includes 406 students from four municipal public educational centers located in the Santiago district of the Metropolitan Region of Chile, with at least 20% migrant enrollment. The data were analyzed using quantitative methods in R Language (R Core Team 2022), with descriptive analysis, cross-tabulation analysis, and the chi-square test. The name of the package used and its reasons for usage are presented in the table below (Table 2):

**Table 2:** R Package, purpose and reason for it’s use.

Package	Purpose	Reason for use
ggplot2 (Wickham, 2016)	Create a variety of graphics and charts in R, such as scatter plots, histograms, box plots, and bar charts	Used for its flexibility and versatility, offering users a lot of control over the look and feel of their visualizations
Tidyverse (Wickham et al., 2016)	A collection of R packages that work together to provide a complete data science toolkit, including data manipulation, visualization, and modeling	Used for its seamless integration with other tidyverse packages, making data manipulation, visualization, and modeling easier and more efficient
Ggstatplot (Patil, 2021)	Provides additional statistical plots and visualizations built on top of ggplot2 that allows users to visualize distributions, correlations, and other statistical relationships in data	Used for its additional statistical plots and visualizations, which made the data analysis and visualization process more efficient and allowed for greater control over the final outputs

Source: Author, 2023.

An analysis of variance (ANOVA) was performed to analyze the relationship between the three ordinal categorical variables of age, status, and educational satisfaction^67^. The variables used in the study were of the Likert type and had a range of values between 1 and 10. Data were collected from a sample of individuals who met the established inclusion criteria, which was selected using simple random sampling to ensure that the results obtained were representative of the population as a whole.

Once the data were collected, they were organized into a contingency table, which included all possible combinations of values for the three categorical variables. This table was used to calculate the frequency of occurrence of each combination of values. With the information obtained in the contingency table, an ANOVA model was constructed that related the three categorical variables.

The ANOVA model allowed predicting the probability of observing a certain value in the dependent variable based on the values of the independent variables, and determined if there was a significant relationship between the variables and if a particular variable was important for predicting a certain outcome^68,69^. According to the data, age has an effect on how satisfied a person is with their education and the relationships they have with their classmates. Therefore, as compared to older students, younger pupils exhibit higher levels of happiness in these areas. Moreover, foreign students showed a statistically significant difference in relation to age range, suggesting that as these students grow older, they tend to become less satisfied with the relationships they form with their classmates. In contrast, no statistically significant difference was found in the Fisher test for Chilean students, indicating that the findings discussed here may not be generalizable to this specific population. The analysis also indicates a significant difference in the entire sample, suggesting a correlation between age range and level of satisfaction with schooling.

To validate the results of the analysis, additional statistical tests were performed, such as the Tukey test, which allows comparing the mean values of the different categories of the dependent variable. "This option should be used when comparing the means of two independent populations (individuals from one of the populations differ from individuals from the other), such as when comparing the populations of men and women "^67^. These tests confirmed the validity of the results obtained and ensured the robustness of the ANOVA model. ANOVA and Tukey's post hoc test were chosen to analyze the relationship between three ordinal categorical variables (gender, age, and educational satisfaction) and overall life satisfaction among adolescent students. ANOVA was used to determine if there was a significant difference in the means of the different groups, while Tukey's post hoc test was used to determine which groups had significantly different means^70^. The chi-square test was used in the cross-tabulation analysis to investigate the relationship between migrant status and age range with the level of satisfaction in forming relationships with peers (tables available on annexes). This was appropriate since both migrant status and age range are categorical variables, and the chi-square test is used to test for significant association between two categorical variables.

In summary, the ANOVA analysis of the three ordinal categorical variables of the Likert type was an effective method for determining the relationship between variables and obtaining accurate predictions about the dependent variable. The use of additional statistical tests allowed confirming the validity of the results and ensuring the robustness of the model, the following (Table 3) describes why the research team used ANOVA, Tukey's post hoc test, and chi-square test in the study:

**Table 3:** Justification of statistical test.

Statistical test	When to use	Explanation
ANOVA	To test for significant differences between means of multiple groups	Appropriate when you want to compare the means of more than two groups to determine if there is a significant difference between them. Can be used for one-way or two-way comparisons
Tukey's post hoc test	To determine which groups have significantly different means after an ANOVA	Appropriate when you have performed an ANOVA and want to perform pairwise comparisons between groups to determine which groups differ significantly from each other
Chi-square test	To test for significant association between two categorical variables	Appropriate when you have categorical data and want to test whether there is a significant relationship between two variables. Can be used for both nominal and ordinal variables

Source: Author, 2023.

### Participants

This study is a quantitative cross-sectional design conducted with adolescent students in 7th and 8th grades of the Chilean educational system. The sample was selected from four municipal public educational centers located in the Santiago district of the Metropolitan Region of Chile. Two criteria were used for selecting schools: convenience and percentage of migrant enrollment, with a requirement of at least 20% migrant enrollment, this is to ensure a proper collection of answers in every school. The sample size was determined based on a 5% error margin and a 95% confidence level, resulting in a total of 406 students evenly distributed across the four participating schools. Among the students, 56.65% were female and 43.35% were male, with 45.81% in 7th grade and 54.19% in 8th grade, and ages ranging from 12 to 16 years with an average of 13.36 years (SD = 0.96). The key variable of the study, native or migrant condition, included 55.91% of students born in Chile and 44.09% of migrants, with similar percentages in terms of school vulnerability index as to avoid major bias in the answers. Among the migrant students, 28.09% were from Peru, 21.35% from Venezuela, 18.54% from Colombia, and 32.02% from other Latin American countries and the rest of the world. The average residence time of migrant students in Chile was 2.59 years (SD = 1.68). For the purposes of this study, students born in Chile were considered as Chilean, and foreign students who had been in Chile for at least 1 year and a maximum of 5 years were considered as migrants. Data were collected through self-administered questionnaires specifically designed to assess variables such as subjective well-being and Overall Satisfaction with life. These instruments were previously adapted and validated for use in the Chilean educational context.

### Instruments

1. Personal Wellbeing Index—School Children 5 (PWI-SC5)^18^

PWI-SC5 is a version (adapted into Spanish) of the Personal Wellbeing Index (PWI^43^), reduced in items and adapted to the school context. PWI was developed with the aim of measuring subjective well-being in the adult population. Subsequently, these authors designed versions for different types of populations, including the Personal Well-Being Index-School Children (PWI-SC) for school-aged children and adolescents^71^. The PWI-SC5 is an adaptation originally for Spanish school settings by Casas et al.^32^, which uses three of the PWI items and adds two additional items at the suggestion of the authors. The items that were used are: satisfaction with all the things I have; my health; my relationships with other people, in general; how adults listen to me; and how I use my time. This instrument uses 11-point scales for responses, ranging from Strongly Disagree (0) to Strongly Agree (10)^72^. Each dimension can be assessed in itself, or an average score can be calculated, in both cases, scores range from 0 to 10. In applications in Chile, the instrument has shown reliability with a Cronbach's Alpha of 0.66 and a good factorial fit (one dimension)^18^. The reliability estimated by Cronbach's alpha coefficient was 0.82, broken down into 0.81 for the subsample of native students and 0.80 for migrant case.

2. Overall Satisfaction with Life (OLS) Cheung and Lucas^73^.

The Overall Satisfaction with Life Scale is an instrument that measures the individual's subjective evaluation of his or her life as an overall whole. This single-item scale ("how satisfied are you with your life, as a whole?") and answered from 0 to 10, ranging from Strongly Disagree (0) to Strongly Agree (10)^73^. It has been applied in children and adolescents as posed showing favorable convergent validity evidence convergent with other measures of satisfaction in Chilean schoolchildren^72^.

It should be noted that participants completed a sociodemographic characterization sheet containing questions regarding gender, nationality, age, grade and time spent in Chile in the case of migrant students.

### Procedure

The self-report questionnaire was applied after the directors of the schools and tutors of the students signed the informed consent. Likewise, the students signed an informed consent form, which was also signed by a teacher of the establishment to guarantee that the students agreed to participate freely. Students, teachers, directors and parents were informed about the confidentiality of the study and the possibility of leaving it at any time without having to give any justification and without any prejudice. The application was developed during the students' school hours during the 2018 school period. The material was given to the students in paper and pencil format, instructions were given and then they were given the time they needed to answer. There was always a responsible teacher and one or more researchers were present in the classroom in order to answer possible questions from the students.

### Aim and objectives of the study

Aim: The aim of this study is to investigate the factors that influence satisfaction in various aspects of academic and social life among foreign and Chilean students in 7th and 8th grade.

## For the sake of this research, we have drawn the following hypotheses:

Hypothesis 1: Age has a significant impact on education satisfaction among foreign students, while it may not be a significant factor for Chilean students.

Hypothesis 2: Foreign students have a decline in satisfaction with schoolmate relationships as they become older than Chilean students do.

Hypothesis 3: Life satisfaction declines with age, foreign students would suffer a greater drop than Chilean students.

Hypothesis 4: Age and immigration status have a major impact on overall life happiness.

Hypothesis 5: Gender has a big impact on student satisfaction with their professors.

### Ethical approval

All the methods were performed in accordance with ethical guidelines. The experimental protocols were approved by the Scientific Ethics Committee of the Universidad de Santiago de Chile. Informed consent was obtained from all subjects and their legal guardians or parents in instances involving minors.

## Results

This study's examination used a conclusive research strategy, concentrating on finding correlations between variables. To explain the links between gender, age, status (migrant or Chilean-born), educational satisfaction, and overall life satisfaction, a descriptive approach was used. The data was analyzed quantitatively in R Language, with the PWI-SC5 measures of subjective well-being and total life satisfaction administered to 406 adolescents (aged 12–16 years). From that sample 56.65% were females and 43.35% were men. The sample included 55.91% Chilean-born students and 44.09% migrant students. Descriptive analysis and cross-tabulation analysis were utilized for data processing, with descriptive analysis providing concise and easily analyzable information^74^. Cross-tabulation analysis was advantageous for interpreting nominal or ordinal data descriptively^74^. However, it should be noted that while a descriptive approach helps in comprehending the acquired data, it might not lead to generalizable conclusions^75^. Additionally, the chi-square test was used in cross-tabulation analysis to examine significant differences between categorical variables for this study.

A summary (Table 4) for the main results is presented below:

**Table 4:** Research questions and key findings.

Research question	Key findings
1. Education satisfaction by age	Younger students express higher levels of satisfaction
	Age has an impact on satisfaction for migrant students
	Age may not significantly impact Chilean students' satisfaction
	Age range correlates with level of satisfaction with schooling
2. Schoolmate relationships by age	Younger students report higher satisfaction
	Age impacts satisfaction with relationships for migrant students
	No statistically significant difference for Chilean students
	Age group correlates with satisfaction with peers
3. Satisfaction with student life	Age range correlates with satisfaction with student life
	Satisfaction tends to decrease as age increases
	Statistically significant relationship for Chilean students
	Limited findings for migrant students
4. Overall life satisfaction	Older individuals report lower levels of satisfaction
	Migrant students become less satisfied with age
	Younger migrant students report the highest satisfaction
	Age correlates with overall life satisfaction for migrant students
5. Satisfaction with teachers by gender	Gender impacts satisfaction with teachers for migrant students
	Male migrant students report higher satisfaction with teachers
	Female migrant students report lower satisfaction with teachers
	Gender correlates with satisfaction with teachers for migrant students

Source: Author, 2023.

Overall, the findings are consistent with those of previous research involving Chile's adolescent demographic, indicating a high level of material satisfaction, similar to other studies focusing on the well-being of adolescents around the age of 12^61^.

### Age-related questions

Education satisfaction varies by age group. Among 12–13 year old foreign students, satisfaction is 83%, while Chilean students report 80.3%. For 14–15 year olds, satisfaction drops to 71.9% for Chileans and 64% for migrants. A significant difference is found for foreign students, suggesting age impacts their satisfaction. No significant difference is found for Chilean students.
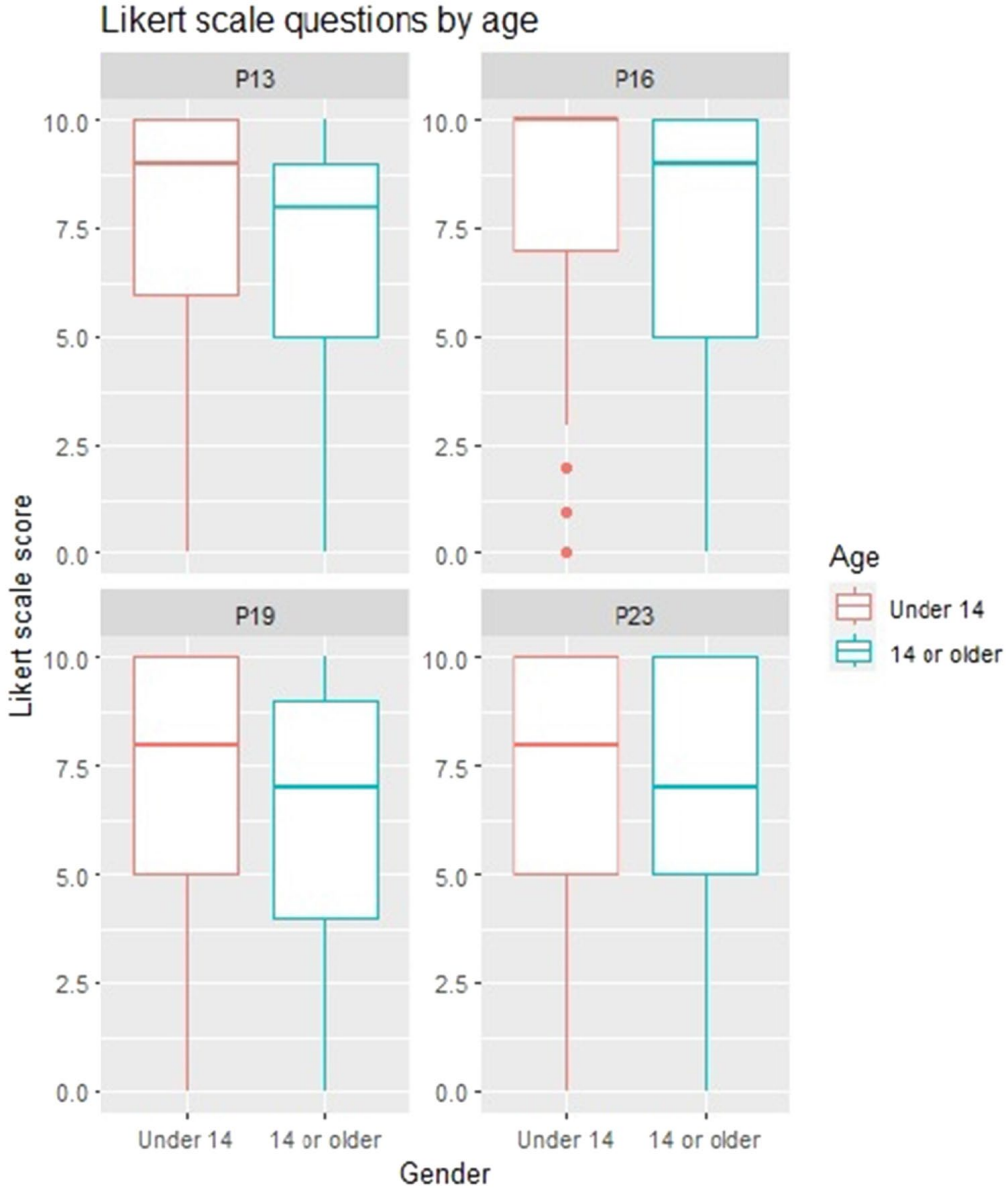


Satisfaction with schoolmate relationships varies by age group. Migrant students aged 12–13 report 80.7% satisfaction, while Chilean students report 84.1%. For 14–15 year olds, satisfaction drops to 68% for migrants and 76.4% for Chileans. Foreign students show a significant difference in relation to age range, suggesting decreased satisfaction, as they grow older. No significant difference is found for Chilean students.

Life satisfaction varies by age group, with differences observed between foreign and Chilean students. Among 12–13 year olds, satisfaction is similar (72.7% for migrants, 73.2% for Chileans). For 14–15 year olds, satisfaction is 65.3% for migrants and 60.7% for Chileans. A significant relationship between age and satisfaction is found for Chileans, but not for migrants.

Overall life satisfaction decreases with age, more pronounced among foreign students. The least satisfied group is migrants aged 14–15, while the most satisfied are migrants aged 12–13. Satisfaction decreases by 13.7% for migrants and 3.9% for Chileans, as they grow older. A significant correlation is found between age and overall life satisfaction for migrant students.

Studies have revealed nuanced differences in satisfaction levels between native and migrant student groups, particularly when examining specific items of the SLSS and BMSLSS scales^76^. Such findings underscore the complex interplay of factors influencing student satisfaction within educational settings.

### Gender-dependent questions

Satisfaction with teachers shows significant differences for male and female foreign students (67.4% for males, 50.6% for females). Male Chilean students report 65.6% satisfaction, while female Chileans report 68%. This highlights the importance of considering gender when analyzing teacher satisfaction.
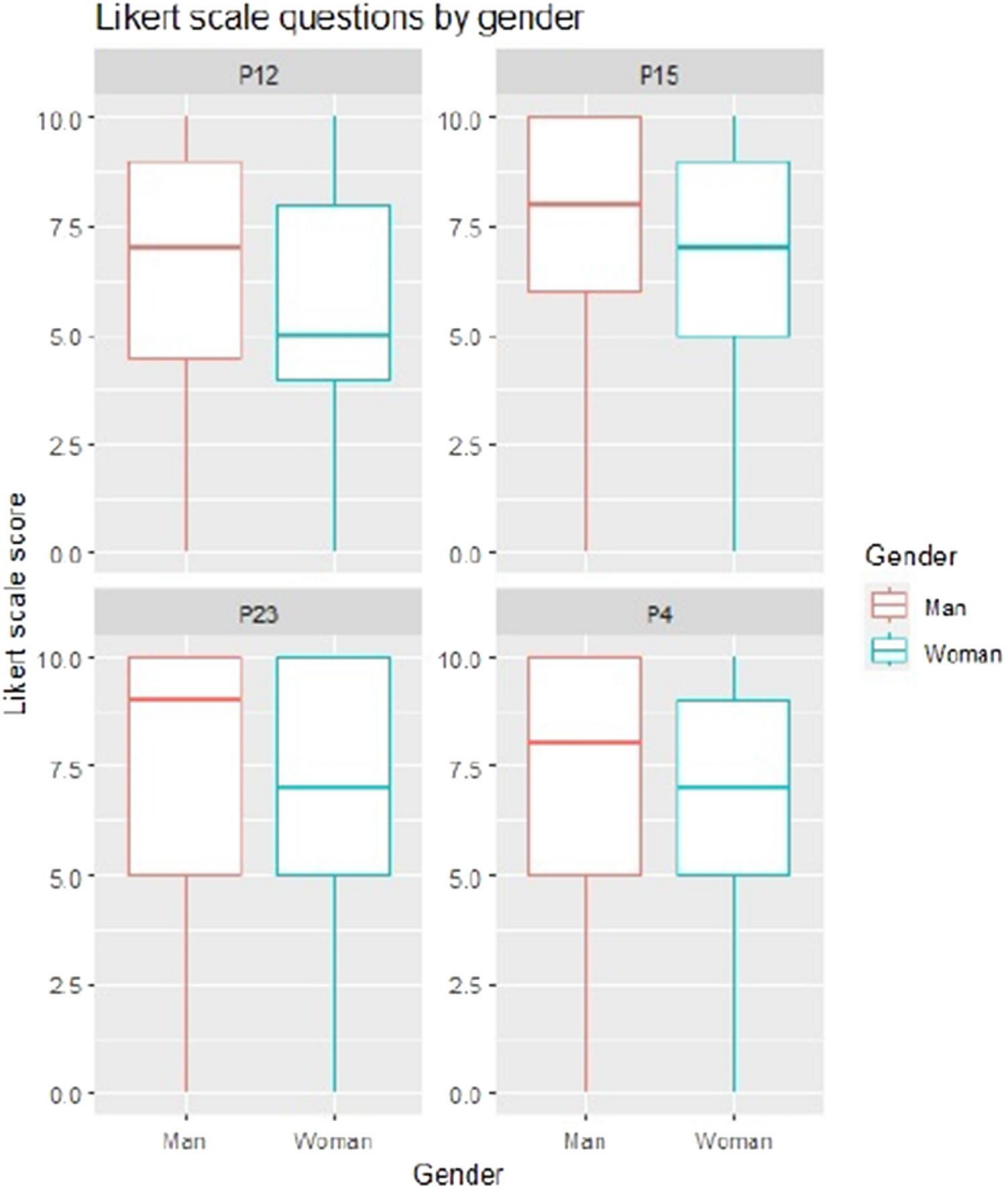


Satisfaction with school performance is lower for female foreign and Chilean students. Male foreign students report 67.4% satisfaction, while female foreign students report 48.1%. Male Chilean students report 54.8% satisfaction, and female Chileans report 48.4%. A significant relationship is found between gender and satisfaction with school performance among international students.

School satisfaction differs between male and female international students, with males reporting 81.4% satisfaction compared to females at 62.3%. Among Chilean students, males score 71%, and females score 67.3%. A significant difference is found in the entire sample, indicating a correlation between gender and overall school satisfaction, particularly among international students.

Overall life satisfaction is lowest for foreign female students (61%), followed by Chilean females (68.6%). Foreign males report 75.6% satisfaction, while Chileans report 72%. A significant gender difference is found in the foreign group, implying a relationship between gender and overall life satisfaction.

### Migration impact on adolescents

Recent research indicates a growing trend of adolescent migration in Chile, marking a significant area of interest for educational studies. Unlike traditional demographics, these young migrants present unique challenges and opportunities within the educational system. This emerging phenomenon necessitates a deeper understanding of their life satisfaction, perceived quality of life, and family self-concept, especially in comparison to their native counterparts^77^

### Hypotheses related results

Hypothesis 1, Age has a significant impact on education satisfaction among migrant students, while it may not be a significant factor for Chilean students is sustained.

Hypothesis 2, Satisfaction with schoolmate relationships decreases, as students grow older, with foreign students experiencing a more pronounced decrease than Chilean students is sustained.

Hypothesis 3, Life satisfaction decreases as students’ age, with the decrease being more significant among migrant students compared to Chilean students is partially sustained as we obtained limited findings for migrant students.

Hypothesis 4, Age and being migrant has a significant effect on overall life satisfaction is sustained.

Hypothesis 5, Gender has a significant impact on satisfaction with teachers is sustained as well.

The current body of research underscores the necessity for more detailed and longitudinal studies to fully grasp the dynamics of student satisfaction across diverse populations. The call for a broader methodological spectrum, including qualitative approaches and the inclusion of varied variables, is essential to capture the rich tapestry of student experiences in a multicultural and migrating educational environment. Such comprehensive research efforts can inform more effective and inclusive educational strategies, ensuring that all students, regardless of their background, can thrive.

## Discussion

This study focuses mainly on the relationship between demographic factors and how they influence student satisfaction in their school life. The study also revealed several patterns and showed some differences between male and female participants.

The results are in agreement with the theoretical framework, which states that the gender and age of adolescents play a crucial role in their satisfaction with their daily life and school life. This concordance in school and daily life satisfaction of students was supported by the data collected, in addition to the coincidence with previous studies showing similar trends in Chile and elsewhere. It is for these reasons that it is worthwhile to inquire more about the causes of these generators of change, whether cultural, social, or any other type of inequality in the development of individuals. Several studies have revealed that elementary school students feel tremendously happy in their school environment. However, this state of happiness diminishes as students grow older and progress through school. For this reason, it is necessary to investigate the causes of this phenomenon and identify the patterns behind it to create strategies that allow both teachers and educational institutions to continue to maintain high levels of satisfaction in students of all ages.

By knowing the expectations that society has of students as they grow older, the requirements they need can be better addressed. In this way, teachers and schools can reverse this situation of dissatisfaction by providing students with greater autonomy and flexibility in their school environment.

Understanding and knowing the causes of dissatisfaction of older students is tremendously important to creating supportive and collaborative networks with society. This would allow us to devise new strategies for older students to develop more fully and satisfactorily, thus enabling them to improve their overall lifestyle.

To generate higher levels of satisfaction in students, it is necessary to make them participate in the decisions that are made, to provide them with constructive criticism, and to lead different types of processes. On the other hand, the cultural background of the students and not only their age should be considered to create environments with higher levels of satisfaction.

As for migrant students and Chilean students, large differences were also found in satisfaction. The levels of dissatisfaction concerning education, relationships with peers, and life in general are high, especially if we refer to older students. The same occurs in terms of gender, where migrants show lower levels of satisfaction than Chilean students. For this reason, it is essential to consider both factors to increase the levels of satisfaction in students (gender and age), especially if we talk about migrants.

The language barrier and cultural differences are also two relevant factors that influence the low level of satisfaction that the migrants may experience. However, there are linguistic support and cultural adaptation programs for these pupils. These factors could be part of the causes of the dissatisfaction experienced by these students. In addition to this, gender-related norms and expectations vary between countries, which adds another problem for male students. There are different ways for teachers and schools to support these students, especially by being aware of and considering cultural differences.

The language barrier and cultural differences are also two relevant factors that influence the low level of satisfaction that the migrants may experience.

A particularly significant factor in increasing levels of happiness among migrant students is the creation of strong and collaborative social networks that allow them to integrate well into their school environment, and also allow them to create relationships with their peers that serve as examples and guidance.

To address the gap in satisfaction levels between migrant and Chilean-born male students, it is necessary to take into consideration cultural differences, gender and its corresponding expectations, age, and any other personal factors of migrants that may be hindering this sense of happiness. Therefore, to create fairer school environments and stronger support networks, the educational community must work together with the external social community.

Research and knowledge advancement: The study of the relationship between migration and the levels of school and life satisfaction in adolescents contributes to expanding the knowledge on issues related to migration, psychology, and education. In addition to expanding the lines of research in this area to have a more comprehensive level of understanding of how migration factors interact with the sense of well-being in these adolescents. With all this information we can work on new strategies and theories.

After identifying the factors that influence greater or lesser school satisfaction in the lives of migrants, educators, and public policymakers can work together to create new norms of coexistence that promote the well-being and integration of these students in the community.

Migration is a window of opportunities to develop inclusive educational policies which can foster the incorporation of highly skilled professional in the future. This is quite possible nowadays given that Chile is well-equipped with a set of scholarship aimed to the more vulnerable groups to pay for their tuition at higher educational levels. These inclusive policies can also help diminish gender disparities in Chilean society and avoid other conflicts derived from the presence of diversity.

In the context of improved educational practices, new teaching techniques that touch on life satisfaction, self-care, socioemotional learning, and wellbeing would be greatly appreciated as a cross-cutting way to enhance school environments and prevent risky behaviors later in adolescence.

This study also makes it possible to examine the potential effects of other dimensions, such as agency and participation, on student satisfaction because increased engagement, empowerment, and involvement in various decision-making processes might enhance the educational experience.

More connections with educational stakeholders, closer ties within the school community, and an increased understanding of the significance of teachers, age, and gender as factors of satisfaction for both native and migrant students can lead to the development of new mentoring skills and long-term positive effects on society.

Incorporating migrants into the classroom can present a chance to enhance cultural competences and leverage diversity to create a more productive and competitive society that can be measured against other international experiences where interculturality has benefited nations.

## Conclusion

The discovery of how demographic, age and gender variables influence the levels of satisfaction in school adolescents in Chile has been of great help to the world of academic research thanks to the knowledge provided by their findings. The differences between Chilean-born and foreign-born students are one of the most important.

This study provided us with the necessary evidence to support our hypothesis that age is a determining factor when it comes to relating satisfactorily with other members of the student community, especially when it comes to foreign students, and that the older they get, the more obstacles they encounter in relating satisfactorily with other members of the student community. This could be due to a natural process of growth in which adolescents tend to individualize and become more independent. However, for foreign students, these challenges may be even greater due to language barriers, cultural differences, and their adaptation to the new culture, in addition to the discrimination they may suffer. For this reason, both educators and policymakers must work together to create new strategies to support older students, especially migrants. To this end, we propose several strategies that can help students have a better school experience. For example, providing counseling services, and peer mentorship programs, as well as creating a school curriculum in which the developmental stages and cultural background of students are considered, as all these new ideas are not considered in school planning in Chile.

The lower level of satisfaction as students get older was also evident. This could occur because of the factors mentioned above that are typical in adolescents, including academic stress, social pressure, and problems they may have with their identity. For foreign students, the factors that influence low satisfaction, in addition to those already mentioned, are added new ones such as adaptation to the new culture, separation from their family of origin, or even legal problems. All these factors lead to seeing the problem more holistically to elaborate new, more integrative, and satisfactory strategies to face the issue, where it is necessary to include the social and emotional needs of the students. For this, all members involved in the school community, whether teachers, principals, parents, among others, must work together for the welfare of students by providing the necessary cultural and mental health support.

Finally, the study shows that gender plays a relevant role in the satisfaction of students and their teachers, especially with foreign students. This means that male students differ from female students in terms of their expectations of their teachers. This is why a new way of doing pedagogy should be considered, from a gender-sensitive perspective, where teachers are aware of the biases that exist between men and women, and where they can have the necessary tools to address these issues and their different edges. In this area, our suggestion is, first, to train teachers in gender issues; second, to use inclusive material; and third, to balance class participation between men and women. All this is part of the basic training that a teacher should have.

In any case, this study has its limitations, since the fact that the samples were taken only in the metropolitan region of Chile means that it is not representative of the entire school population of the country, nor migrants. Therefore, no causal relationship can be inferred from the correlations observed in this cross-sectional study.

This study allows us to visualize interesting topics for further research but using a longitudinal design where it is possible to observe changes in student satisfaction over the years. In addition, it would be interesting to expand the range of research to measure satisfaction by considering other factors such as school environment, teacher methodology, and personality traits.

Despite the limitations of this study, however, it has enough positive effects to generate new educational policies and practices. As we increase our understanding of the different groups involved, we can continue to foster more inclusive and supportive learning environments, especially with migrant students. These policies and strategies that would consider their culture of origin, language, and social needs could provide them with a better and more satisfying educational and life experience.

In conclusion, this study reinforces the relationship between age, gender, and migratory status of the students with the levels of school and life satisfaction. We hope that our findings will allow those of us who are dedicated to research to continue investigating this topic to generate more inclusive strategies for the good of the educational community.

### Supplementary Information

Supplementary Information.

## Data Availability

The data utilized in this study are included as supporting files accompanying the journal submission. Interested researchers may refer to these files for access to the data, or directly contact either Dr. Céspedes or Dr. Leigh.
