# Author Correction: The roles of age, gender, and migration in shaping adolescent student satisfaction within Chilean schools

**DOI:** 10.1038/s41598-024-69713-9

**Published:** 2024-08-19

**Authors:** Cristian Céspedes, Camila Leigh, Enrique Leigh, Peodair Leihy, Sergio Fuentealba-Urra, Andrés Rubio, Damarys Roy

**Affiliations:** 1 Andres Bello University, Faculty of Economics and Business, Santiago, Chile; 2 Centro de Investigación Urbana para el Desarrollo, el Hábitat y la descentralización (CIUDHAD), Universidad Andrés Bello, Santiago, Chile; 3 Universidad Del Desarrollo, Santiago, Chile; 4 Diego Portales University, Faculty of Psychology, Santiago, Chile

Correction to: *Scientific Reports* 10.1038/s41598-024-61427-2, published online 17 June 2024

The original version of this Article contained an error in Affiliation 1, which was incorrectly given as ‘Universidad Andrés Bello, Santiago, Chile.’ The correct affiliation is listed below:

Andres Bello University, Faculty of Economics and Business, Santiago, Chile.

Additionally, an affiliation was omitted for Andrés Rubio. Their correct affiliations are listed below.

Andres Bello University, Faculty of Economics and Business, Santiago, Chile.

Diego Portales University, Faculty of Psychology, Santiago, Chile.

The original Article has been corrected.

