# Supplementary material for: The roles of age, gender, and migration in shaping adolescent student satisfaction within Chilean schools
Source: Sci Rep. 2024 Jun 17;14:13944. doi: 10.1038/s41598-024-61427-2 (PMC11183043; doi:10.1038/s41598-024-61427-2)

Annexes

[Supporting files]

**[Metadata]**

Age-related questions

Question Level of education satisfaction


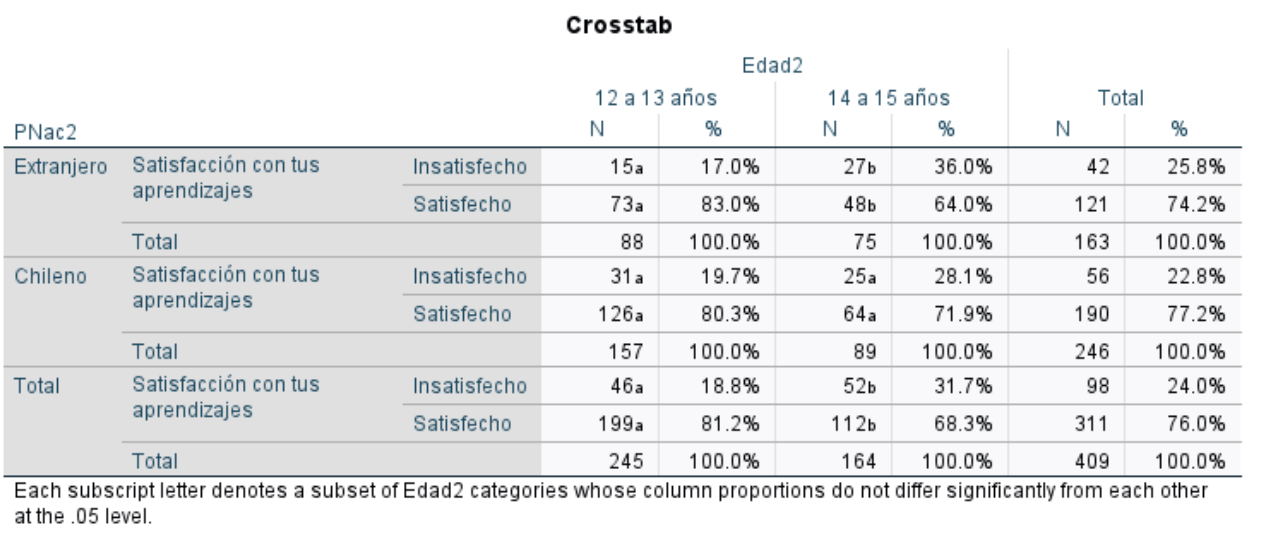


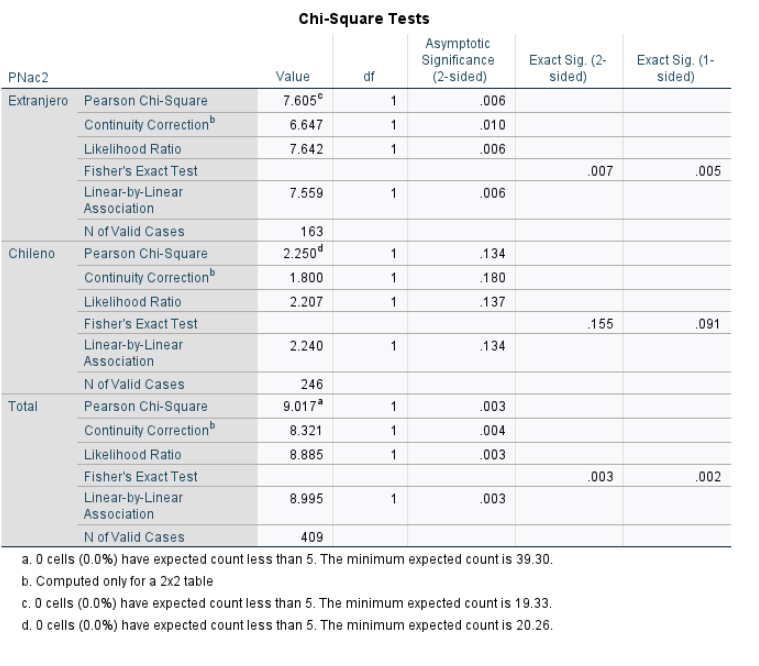


Question Satisfaction with your schoolmates' relationships


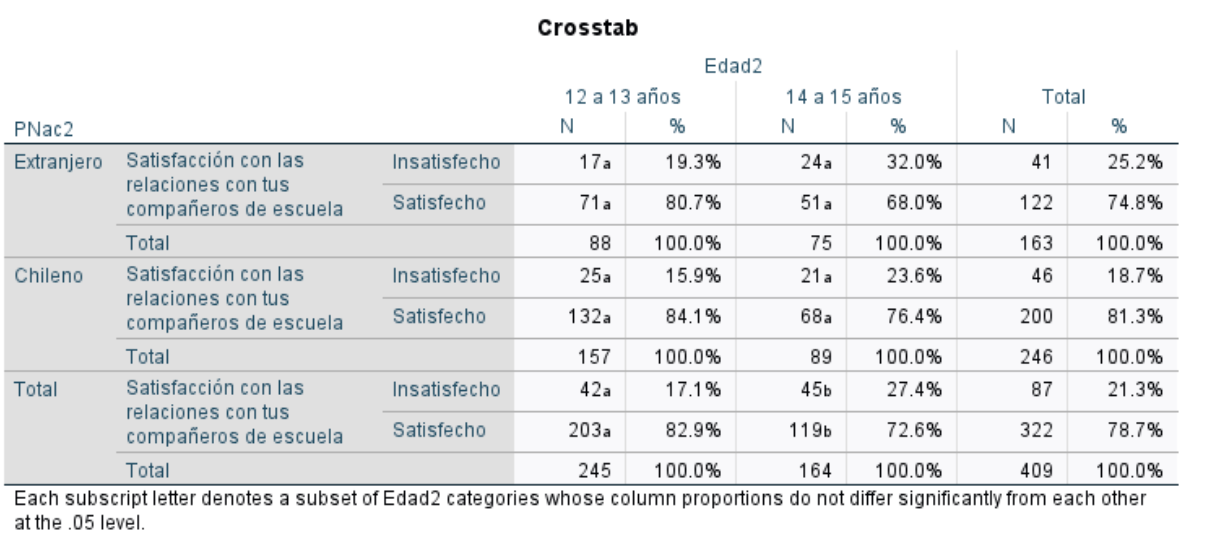


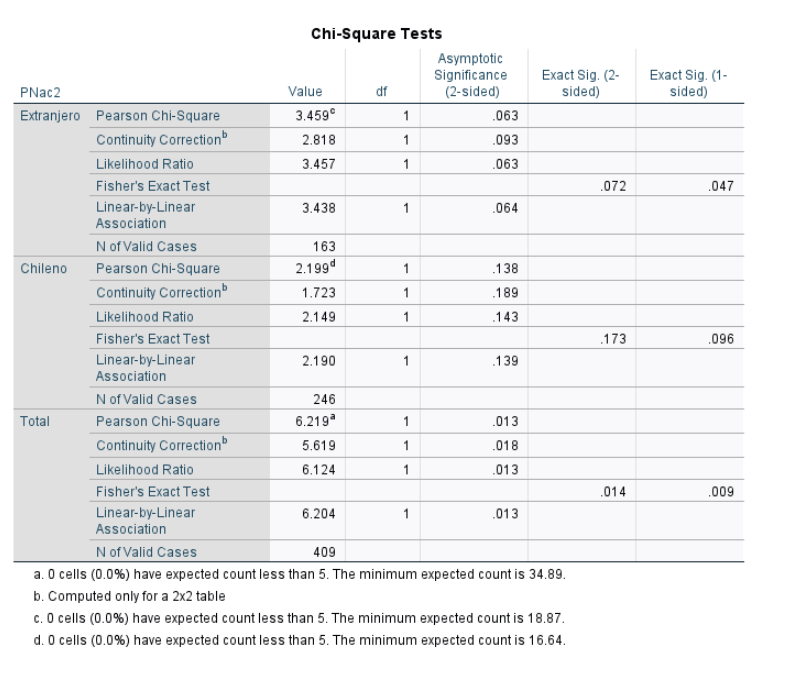


Question Satisfaction with your life as a student


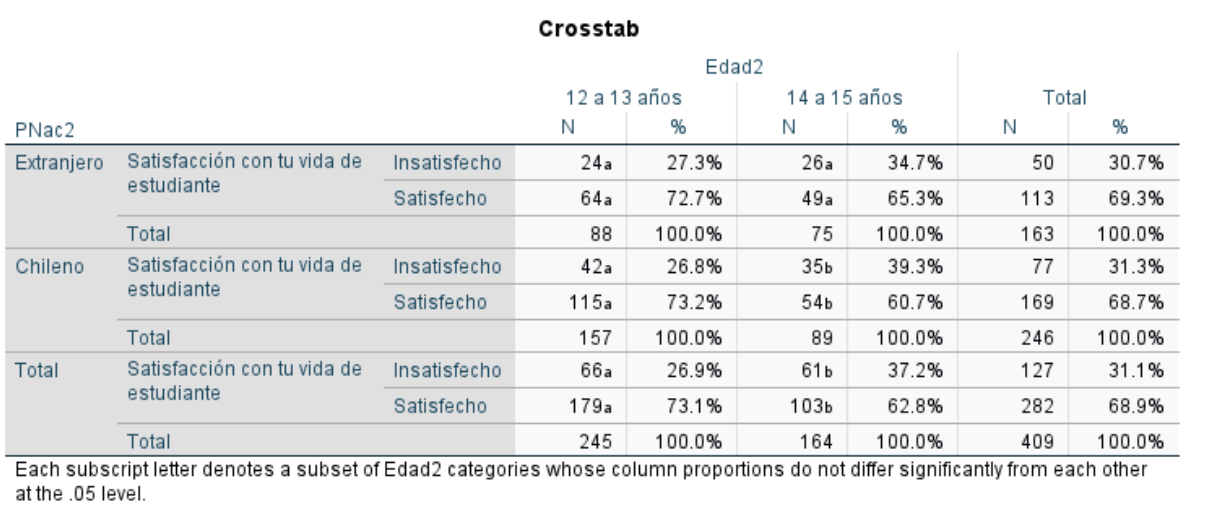


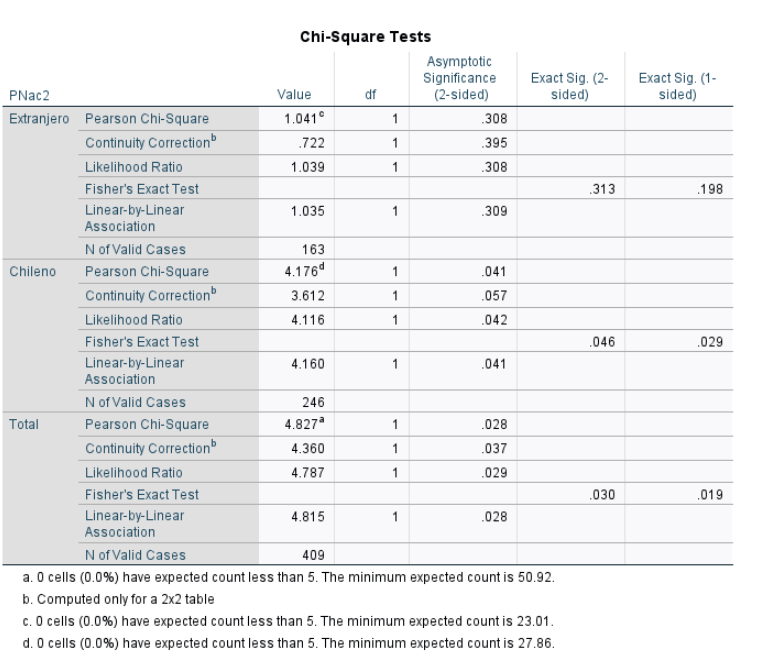


Question Overall life satisfaction


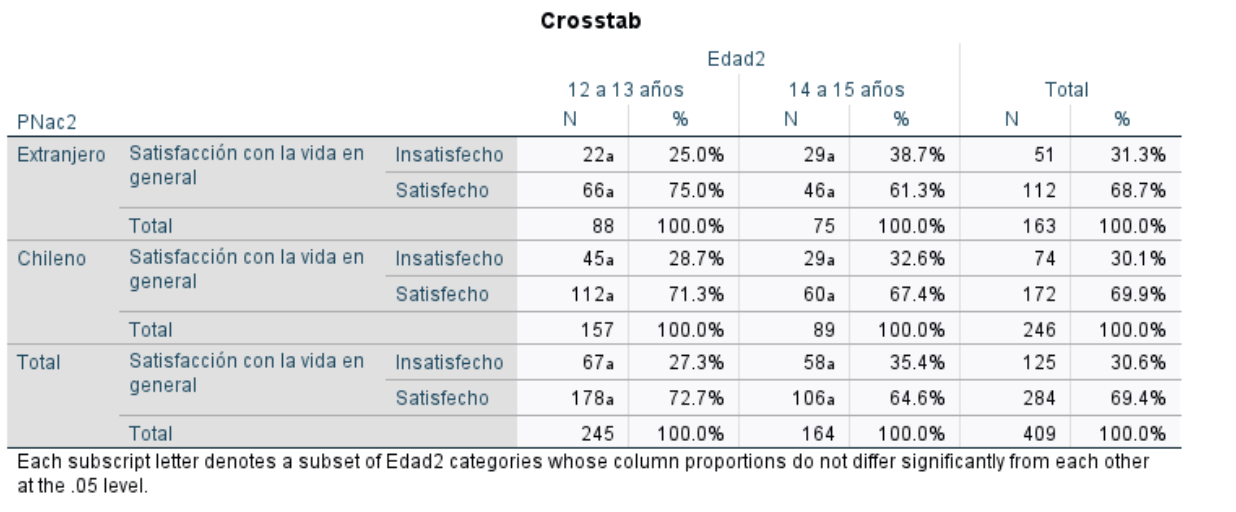


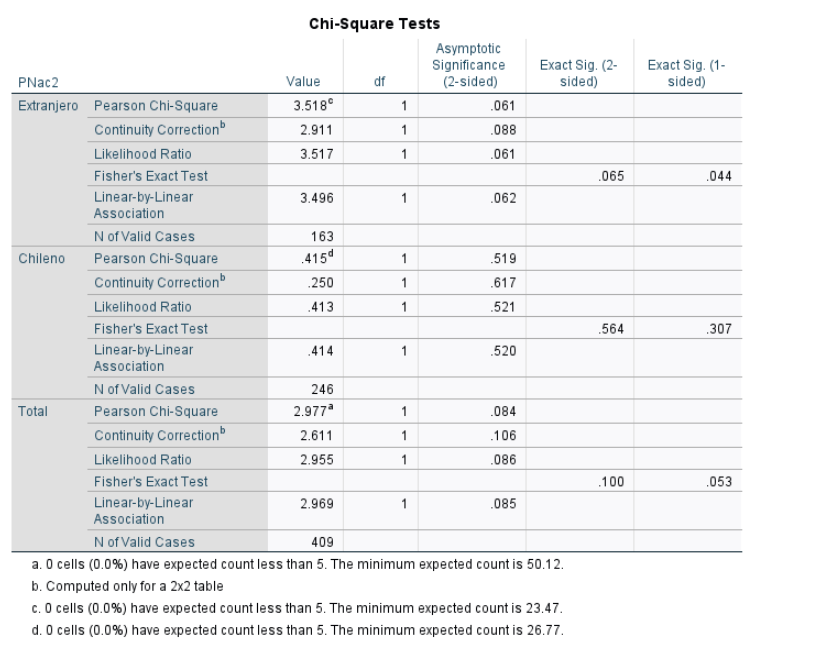


Gender-dependent questions

Question Satisfaction with your teachers


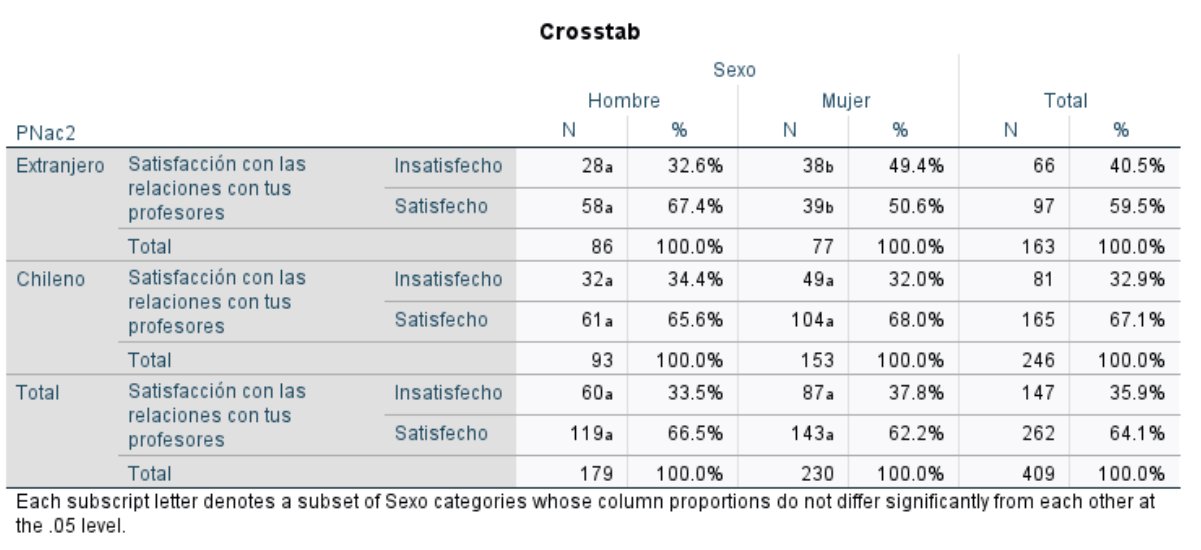


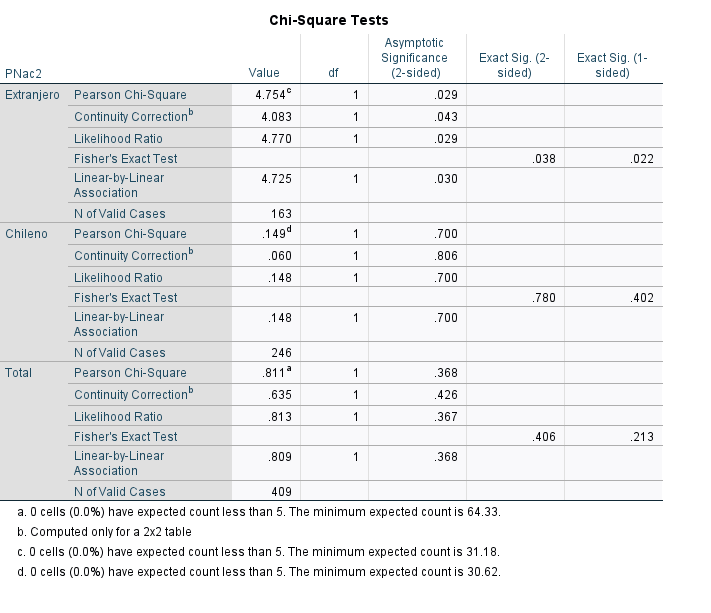


Question Satisfaction with school performance


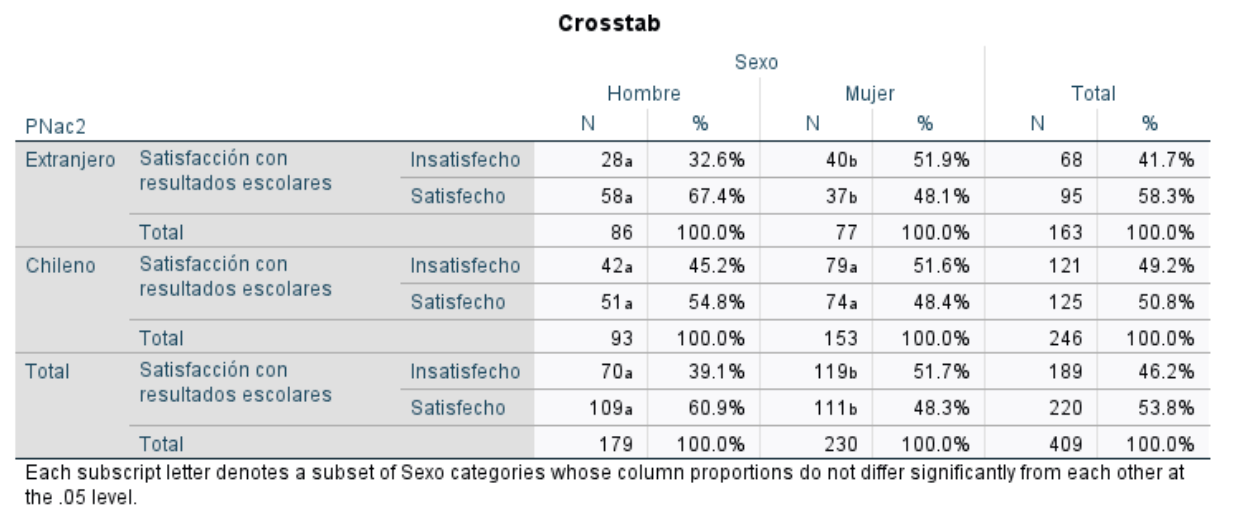


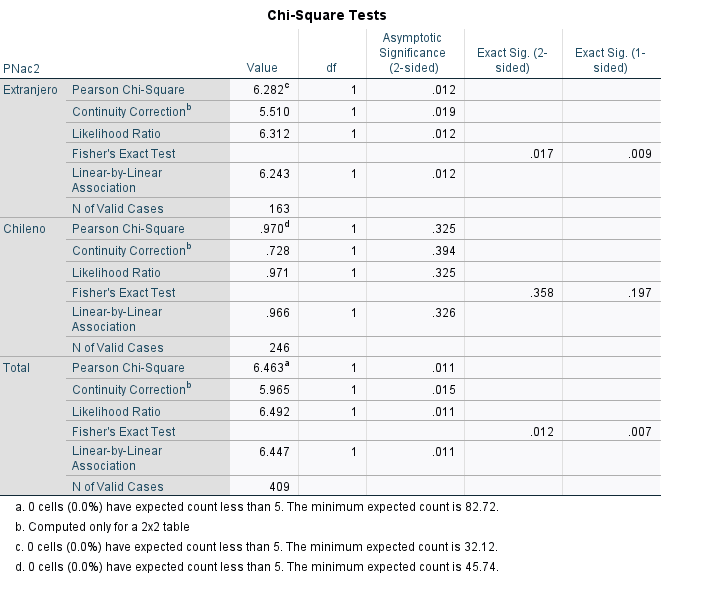


Question: Overall satisfaction with your school


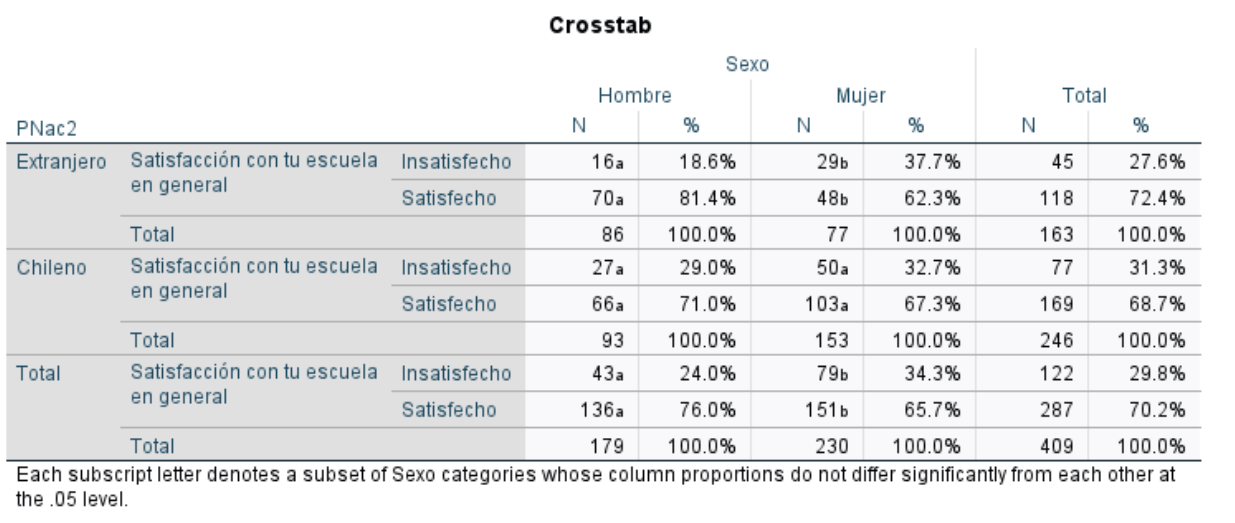


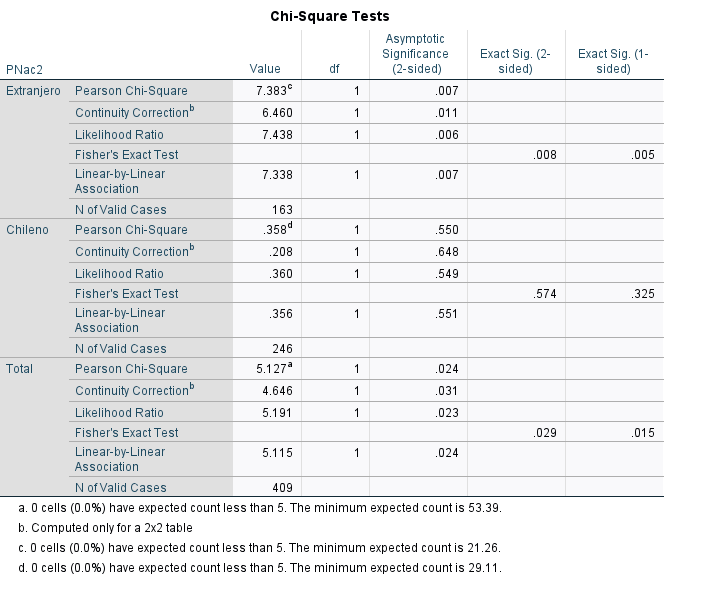


Quiestion: Overall life satisfaction


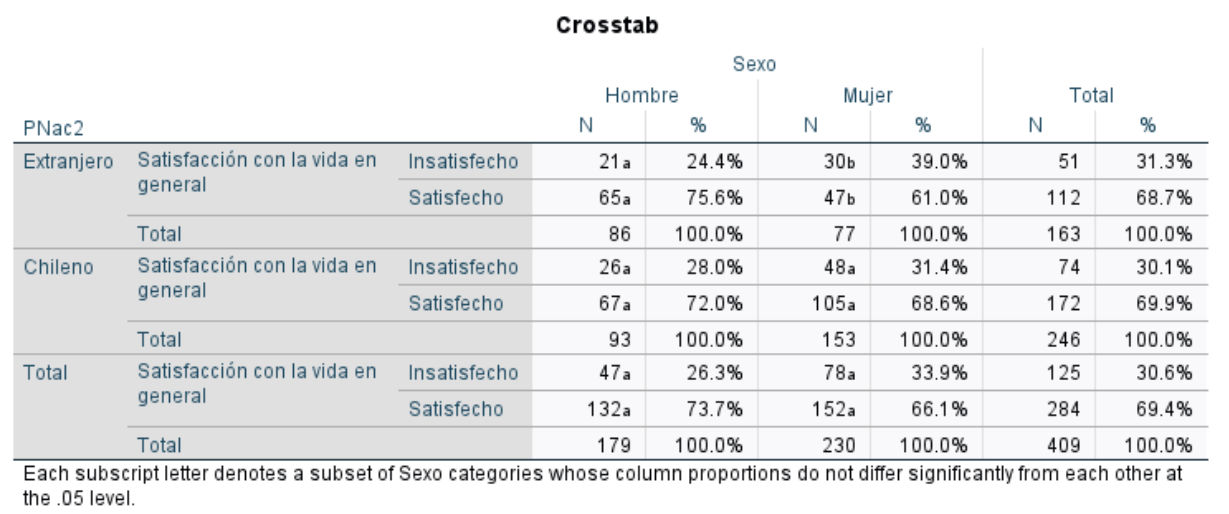

Supplement: Supplementary file 1 — Supplementary Information. [file 41598_2024_61427_MOESM1_ESM.docx]
